# Enhanced Endothelin A and B Receptor Expression and Receptor-Mediated Vasoconstriction in Rat Mesenteric arteries after Lipopolysaccharide Challenge

**DOI:** 10.1155/2019/6248197

**Published:** 2019-11-14

**Authors:** Wei Zhang, Shan-Shan Zhang, Hong-Lang Huang, Bing-Jie Song, Xiao Liu, Zhi Qi

**Affiliations:** ^1^Xiamen Institute of Cardiovascular Diseases, The First Affiliated Hospital of Xiamen University, School of Medicine, Xiamen University, Xiamen 361003, China; ^2^Department of Basic Medicine, School of Medicine, Xiamen University, Xiamen 361102, China

## Abstract

During organ culture of intact vessels, endothelin receptors (ETRs) were upregulated in vascular smooth muscle cells (VSMCs) by various stimuli, but whether inflammation alters ETR expression in vivo remains unclear. We aimed to explore the effects of lipopolysaccharide (LPS) challenge on ETR expression in the VSMC in vivo. Male Sprague-Dawley rats received a single intraperitoneal injection of LPS (5 mg/kg body weight) or normal saline (NS) for 6 hrs. The function and expression of ETR type A (ET_A_) and type B (ET_B_) were evaluated in the mesenteric arteries without endothelium, by using myograph system, real-time quantitative PCR, Western blot, and immunohistochemical staining, respectively. Serum tumor necrosis factor-*α* (TNF-*α*) level was assessed by using enzyme-linked immunosorbent assay. The results showed that, compared to control (NS) group, LPS treatment potently enhanced the vasoconstriction mediated by ET_A_ or ET_B_ in rat mesenteric artery, with elevated maximum effects. ET_A_ and ET_B_ expressions in the VSMC were increased at both mRNA and protein levels after LPS treatment, paralleled with activation of the NF-*κ*B pathway and augmented serum TNF-*α* level. Conclusively, in the rat model of immediate systemic inflammation induced by LPS, ET_A_ and ET_B_ expressions were increased in the mesenteric arterial VSMC, paralleled with enhanced receptor-mediated vasoconstriction and activation of the NF-*κ*B pathway. Our data has for the first time demonstrated the upregulation of ETRs in VSMCs by LPS-induced immediate inflammation in vivo.

## 1. Introduction

Vasospasm and vascular wall remodeling are important functional/morphological disorders in the vascular wall. Numerous stimuli target on vascular smooth muscle cells (VSMCs) and alter vasomotion, being major pathological factors in the progression of atherosclerotic vascular diseases. In the vasculature, endothelin-1 (ET-1) is one of the most potent endogenous vasoconstrictors, showing various biological functions through binding to its receptors. Among the subtypes of endothelin receptors (ETRs), endothelin type A receptor (ET_A_) is expressed on VSMC, mediating vasoconstriction and proliferation [1, 2], while endothelin type B receptor (ET_B_) is normally expressed on vascular endothelial cells (VECs), mediating vasodilatation of VSMC via nitric oxide (NO) and prostacyclin I_2_ (PGI_2_) pathways [3]. Distinct from such relaxing phenotype of ET_B_ (termed ET_B1_), a contractile phenotype of ET_B_ (termed ET_B2_) was discovered on VSMC, mediating vasoconstriction under certain pathophysiological conditions. Abundant studies reported aberrant elevation of ETRs, specifically upregulated ET_A_ and induced ET_B2_, in the VSMC in animal models and human subjects with various cardiocerebrovascular diseases [4]. Augmented expressions of ET_A_ and ET_B2_ result in enhanced vasoconstriction triggered by their endogenous ligand, ET-1, and thus lead to modulated vascular tone and accelerated proliferation of VSMC.

It has been well-documented that inflammation plays a crucial role in the progression of cardiovascular diseases. Dysregulation of serum proinflammatory cytokines elicits deleterious effects to the VSMC via activation of intracellular signals and therefore causes vascular dysfunction. Previously, by using an in vitro organ culture model, we demonstrated the upregulation of ET_B2_ expression in VSMC through the activation of the nuclear factor-*κ*B (NF-*κ*B) pathway after exposure of the arteries to tumor necrosis factor-*α* (TNF-*α*), a key proinflammatory cytokine [5]. Additionally, during organ culture, expressions of ET_A_ and ET_B2_ were enhanced by lipid-soluble smoking particles [6, 7] and low-density lipoprotein (LDL) [8]. The above data, to some aspect, indicated a link between upregulation of ETRs and inflammation associated with cardiovascular risk factors. Nevertheless, there is still a lack of in vivo data concerning the altered expression of ETRs in mesenteric arterial VSMC in an efficient animal model of inflammation. Intraperitoneal administration of lipopolysaccharide (LPS) could increase TNF-*α* production by peritoneal macrophages and is thus widely employed as an animal model of immediate systemic inflammation [9, 10]. To further verify the induction of ETRs by inflammation in vivo, we herein established a classic rat model of LPS-induced immediate systemic inflammation, and the mesenteric arteries were used for the further study.

## 2. Materials and Methods

### 2.1. Animals and Tissue Preparation

Male Sprague-Dawley rats (SPF, weighing about 200 g) were purchased from Shanghai Center of Experimental Animals, Chinese Academy of Sciences (Shanghai, China). Rats had free access to water and standard rat chow pellets and were housed under controlled temperature (22 ± 1°C) and humidity (50-60%) with a 12 hr light-dark cycle from 7 AM to 7 PM. After acclimatization for 1 week, thirty rats were randomly allocated into two groups: normal saline (NS) and LPS, receiving a single intraperitoneal administration of equivalent volume of NS or LPS (5 mg/kg body weight), respectively. LPS (Sigma-Aldrich, Saint Louis, MO, USA) was dissolved in normal saline. Six hours after injection, rats were anesthetized with an intraperitoneal injection of pentobarbital sodium (10 mg/rat). The blood was drawn from carotid arteries of rats using a catheter (24G), centrifuged at 2000 rpm for 15 min within 30 min of collection and stored at -80°C until assayed. After blood collection, euthanasia of rats was performed by decapitation. The mesenteric tissue sample was gently removed from the abdomen. Dissection of the mesenteric artery and denudation of the endothelium with Triton X-100 were performed as described previously [6]. All the animal experimental procedures were approved by the Ethics Committee on Animal Research from The First Affiliated Hospital of Xiamen University, complying with Animal Research: Reporting of In Vivo Experiments (ARRIVE) Guidelines, and were carried out in accordance with the National Institutes of Health Guide for the Care and Use of Laboratory Animals (8th Edition) and American Veterinary Medical Association (AMVA) Guidelines for the Euthanasia of Animals (2013 Edition).

### 2.2. Functional Assay (Myograph)

The arteries (without endothelium) were cut into 1 mm long cylindrical segments and mounted to a myograph system (620 M, Danish Myo Technology A/S, Aarhus, Denmark) for recording the receptor-mediated vasoconstriction. The concentration-response curves (CRCs) were performed by cumulative administration of selective ET_B_ agonist sarafotoxin 6c (S6c, Sigma-Aldrich, Saint Louis, MO, USA), followed by nonselective ETR agonist ET-1 (Calbiochem, La Jolla, CA, USA), as previously described [6, 11]. Briefly, after S6c CRCs were obtained, the arterial rings were coincubated with S6c (10^−7.5^ M) for 30 min. The desensitization of ET_B_ was verified by lack of response to further administration of S6c (10^−7^ M). The subsequent ET-1 CRCs represent ET_A_-mediated vasoconstriction. This method is comparable to the application of BQ-788, the selective ET_B_ antagonist, for assessment of ET_A_-mediated vasoconstriction [12]. S6c and ET-1 were dissolved in bovine serum albumin solution (0.1%, Sigma-Aldrich, Saint Louis, MO, USA).

### 2.3. RNA Extraction and Real-Time Quantitative Reverse Transcription Polymerase Chain Reaction (QRT-PCR)

The arterial segments (without endothelium, 6 mm in length) were homogenized in Lysing Matrix D centrifuge tubes (MP Biomedicals, Santa Ana, CA, USA), containing extraction buffer obtained from RNeasy Mini Kit (Qiagen, Hilden, Germany), in a FastPrep-24 5G homogenizer (MP Biomedicals, Santa Ana, CA, USA). Total RNA was extracted following the manufacturer's instructions. Reverse transcription of total RNA to cDNA was carried out with SuperScript III First-Strand Synthesis System (Invitrogen, Carlsbad, CA, USA) in a 2720 Thermal Cycler (Applied Biosystems, Carlsbad, CA, USA) following the manufacturer's instructions.

Real-time quantitative PCR was performed in a QuantStudio 6 Flex Real-Time PCR system (Applied Biosystems, Carlsbad, CA, USA) with the reaction protocol as described previously [6]. Primers were as follows: ET_A_ (Ednra, GenBank accession no. NM_012550) mRNA: forward 5′-GCGTCGAGAGGTGGCAAA-3′ and reverse 5′-CCAGCACAGGGCGAAGAT-3′; ET_B_ (Ednrb, GenBank accession no. NM_017333) mRNA: forward 5′-GATACGACAACTTCCGCTCCA-3′ and reverse 5′-GTCCACGATGAGGACAATGAG-3′. Elongation factor-1 (EF-1, Eef1a1, GenBank accession no. NM_175838) mRNA was used as reference (internal control) [5, 11]. The primers were forward 5′-GCAAGCCCATGTGTGTTGAA-3′ and reverse 5′-TGATGACACCCACAGCAACTG-3′. All the primers were synthesized by Sangon Biotech (Shanghai, China). Relative expressions of ETR mRNA were analyzed with 2^-ΔΔCt^ method and normalized with EF-1 mRNA expression.

### 2.4. Hematoxylin-Eosin and Immunohistochemical Staining

Paraformaldehyde-fixed and paraffin-embedded arterial segments (without endothelium, 6 mm in length) sections were used for hematoxylin-eosin and immunohistochemical staining. Briefly, after fixation with 4% neutral formaldehyde and washed with running water and 75% ethanol. After dehydrated and hyalinized in a TP1020 tissue processor (Leica, Nussloch, Germany), tissues were then embedded by a HistoCore Arcadia (Leica, Nussloch, Germany) and cut at 3.5 *μ*m thickness with an RM2245 semimotorized rotary microtome (Leica, Nussloch, Germany). Dewaxing of the tissues was performed with Van-Clear reagent (Huntz, Wuhan, China) and gradient ethanol. Sections were then used for downstream staining with hematoxylin-eosin or primary antibodies. The following antibodies were used: rabbit polyclonal antibodies against ET_A_ (ab117521, 1 : 2000, Abcam, Cambridge, UK) and ET_B_ (ab117529, 1 : 2000, Abcam, Cambridge, UK) and horseradish peroxidase-conjugated goat anti-rabbit secondary antibody (A16110, 1 : 200, Invitrogen Carlsbad, CA, USA). Sections were then visualized with a DAB kit (Maixin Biotech, Fuzhou, China), counterstained with hematoxylin, and observed under an Echo Revolve hybrid microscope (Echo Laboratories, San Diego, CA, USA). No detectable staining was observed in all negative control slides treated with nonimmune rabbit serum (10%) (Santa Cruz Biotechnology, Santa Cruz, CA, USA). The experiments were repeated 3 times independently.

### 2.5. Western Blot

Total protein was extracted from the arterial segments (without endothelium, 9 mm in length) in Lysing Matrix D centrifuge tubes (MP Biomedicals, Santa Ana, CA, USA), containing T-PER Tissue Protein Extraction Reagent (Pierce, Rockford, IL, USA) supplemented with Halt Protease and Phosphatase Inhibitor Cocktail (Pierce, Rockford, IL, USA), in a FastPrep-24 5G homogenizer (MP Biomedicals, Santa Ana, CA, USA). Protein concentration was measured using BCA Protein Assay Kit (Pierce, Rockford, IL, USA). Proteins (15 *μ*g) were loaded and separated in NuPAGE Novex 10% Bis-Tris Gel (Invitrogen, Carlsbad, CA, USA) and transferred to polyvinylidene difluoride membrane (Millipore, Bedford, MA, USA). The membrane was immersed in blocking buffer (0.05% Tween-20 and 3% non-fat milk in phosphate buffered saline) on a shaker at room temperature for 1 hr, followed by incubation in primary antibody at 4°C overnight. After washing with phosphate buffered saline containing 0.05% Tween-20 for 3 times, the membrane was incubated with secondary antibody at room temperature for 1 hr and then washed.

The membrane was visualized by using WesternBright ECL HRP Substrate (Advansta, Menlo Park, CA, USA) and captured in a c600 fluorescence and chemiluminescence imager (Azure Biosystems, Dublin, CA, USA). The primary antibodies for ET_A_ (ab117521, 1 : 2000), ET_B_ (ab117529, 1 : 2000), phospho-NF-*κ*B p65 (ab86299, 1 : 2000), NF-*κ*B p65 (ab16502, 1 : 2000), and the reference (internal control) *β*-actin (ab8226, 1 : 5000) were purchased from Abcam (Cambridge, UK). Horseradish peroxidase-conjugated secondary antibodies (goat anti-mouse, A16078, 1 : 10000; goat anti-rabbit, A16110, 1 : 10000) were from Invitrogen (Carlsbad, CA, USA). The experiments were repeated 3 times independently.

### 2.6. Enzyme-Linked Immunosorbent Assay (ELISA)

A solid-phase sandwich ELISA kit for rat TNF-*α* (Invitrogen, Camarillo, CA, USA) was used to determine the serum TNF-*α* level in rats, following the manufacturer's instructions.

### 2.7. Statistical Analysis

All data are expressed as mean ± standard deviation (SD). The vasoconstrictions are presented as the percentage of response to 60 mM K^+^. The relative mRNA expression of ETR is presented as a fold change of control (NS) group, normalized with the mRNA expression of housekeeping gene EF-1. All data passed normality tests (*α* = 0.05). Unpaired Student's *t*-test was used for comparisons of two data sets. Two-way analysis of variance with Bonferroni post-test was used for comparisons of series (functional) data. A *P* value less than 0.05 was considered to be significant.

## 3. Results

### 3.1. ETR-Mediated Vasoconstriction of Mesenteric Arteries Was Enhanced by LPS Treatment

**Figure 1:**
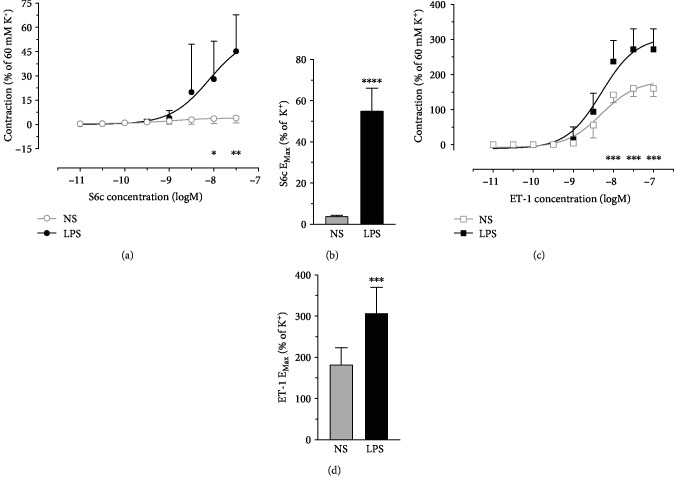
Functional assay (myograph) experiments showed enhanced CRCs of vasoconstriction mediated by (a) ET_B_ and (C) ET_A_ and increased *E*_Max_ of agonists for (b) ET_B_ and (d) ET_A_ in VSMC of mesenteric arteries in rats received LPS (5 mg/kg body weight) injection for 6 hrs. Data are expressed as mean ± SD. Two-way ANOVA with Bonferroni post-test (CRC) or unpaired Student's *t*-test (*E*_Max_), ^∗^*P* < 0.05, ^∗∗^*P* < 0.01, ^∗∗∗^*P* < 0.001, ^∗∗∗∗^*P* < 0.0001 versus NS, *n* = 6 (NS) or 4 (LPS) animals for each data point.

To study the vasoconstriction mediated by ETR subtypes, arteries were mounted to a myograph system, and selective ETR agonists were cumulatively added to the organ bath. CRCs mediated by ET_B_ (S6c) and ET_A_ (ET-1 following desensitization of ET_B_) were potently elevated after LPS injection to rats, with increased maximum effects (*E*_Max_) of the agonists (Figure 1). The calculated pEC_50_ values (NS versus LPS) were S6c 9.027 ± 0.08561 versus 8.106 ± 0.1287 and ET-1 8.299 ± 0.1457 versus 8.299 ± 0.137, respectively.

### 3.2. ETR Expression in VSMC Was Augmented by LPS Treatment

**Figure 2:**
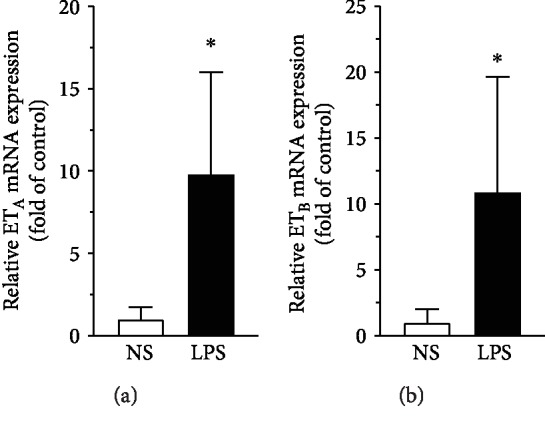
Induction of (a) ET_A_ and (b) ET_B_ mRNA expressions in VSMC in rat mesenteric arteries by LPS injection assessed by real-time QRT-PCR. Data are expressed as mean ± SD. Unpaired Student's *t*-test, ^∗^*P* < 0.05 versus NS, *n* = 5 animals in each group.

**Figure 3:**
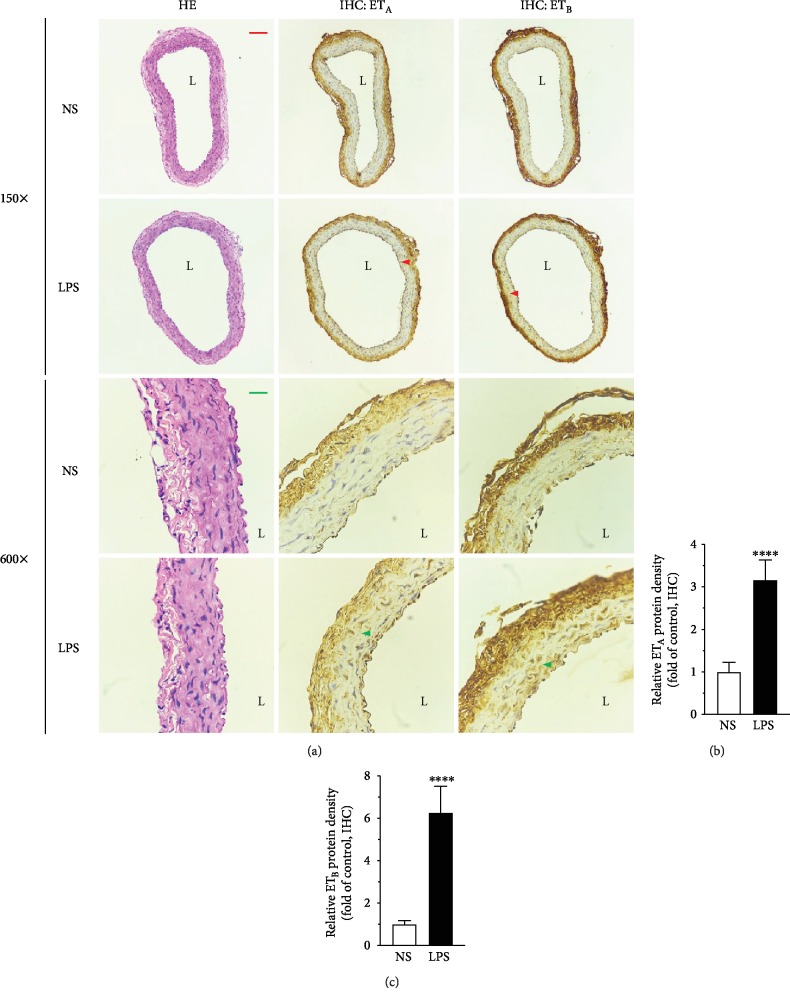
Hematoxylin-eosin staining and immunohistochemical staining on paraffin-embedded tissue sections showed the morphology of the vessels and the upregulation of ET_A_ and ET_B_ after LPS treatment, respectively. Arteries (without endothelium) were taken from rats treated with NS or LPS (5 mg/kg body weight) for 6 hrs. The arrowhead points to the positive staining of ET_A_ or ET_B_ protein. L: lumen. The size bar corresponds to 200 *μ*m (150x) or 50 *μ*m (600x. (b, c) Semiquantitation of immunohistochemistry results by using ImageJ software. Data are expressed as mean ± SD. Unpaired Student's *t*-test, ^∗∗∗∗^*P* < 0.0001 versus NS, *n* = 10 sections (2 sections per animal) in each group.

**Figure 4:**
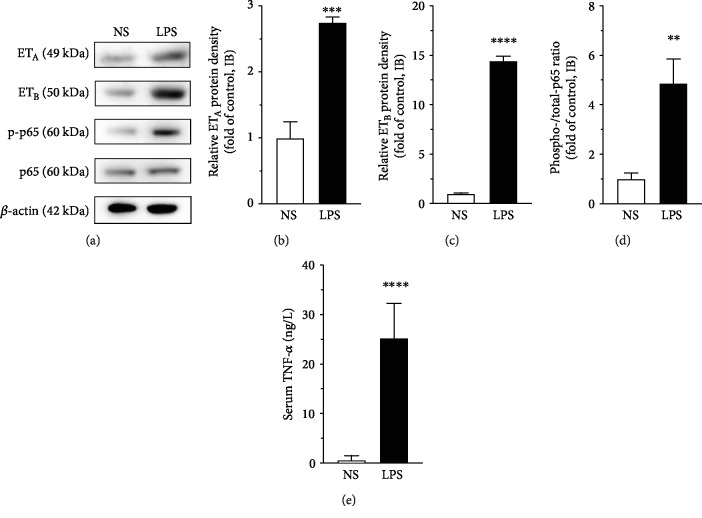
(a) Western blot demonstrated enhanced protein expression of ET_A_ and ET_B_, and phosphorylation of NF-*κ*B p65 in VSMC in rat mesenteric arteries after LPS injection. The immunoblot of *β*-actin was used as reference (internal control). (b–d) Semiquantitation of Western blot results by using ImageJ software. Data are expressed as mean ± SD. Unpaired Student's *t*-test, ^∗∗^*P* < 0.01, ^∗∗∗^*P* < 0.001, ^∗∗∗∗^*P* < 0.0001 versus NS, *n* = 3 animals in each group. (e) Serum TNF-*α* was increased by LPS (5 mg/kg body weight) injection for 6 hrs in rats measured by ELISA. Data are expressed as mean ± SD. Unpaired Student's *t*-test, ^∗∗∗∗^*P* < 0.0001 versus NS, *n* = 5 animals in each group.

To evaluate the mRNA and protein expression of ET_A_ and ET_B_ in the VSMC in mesenteric arteries, QRT-PCR, immunohistochemistry, and Western blot were performed. Compared to that in the control (NS) group, LPS treatment remarkably induced both the mRNA (Figure 2, *P* < 0.05) and the protein (Figure 3, *P* < 0.0001; Figures 4(a)–4(c), *P* < 0.001) expression of ET_A_ and ET_B_ in the VSMC.

### 3.3. LPS Treatment Activated the NF-*κ*B Pathway in the VSMC

The phosphorylation of NF-*κ*B p65, the key enzyme of the NF-*κ*B pathway, was detected by using Western blot. LPS treatment significantly enhanced the phosphorylation of NF-*κ*B p65 (phospho/total p65, Figures 4(a) and 4(d), *P* < 0.01), indicating the activation of the NF-*κ*B pathway in the VSMC in rats treated with LPS.

### 3.4. LPS Treatment Increased Serum TNF-*α* Level in Rats

ELISA results showed that, 6 hrs after intraperitoneal injection of LPS (5 mg/kg body weight), the serum level of TNF-*α* was significantly increased in rats (Figure 4(e), *P* < 0.0001), indicating an immediate systemic inflammatory response in the animals.

## 4. Discussion

Vital roles of ETRs in embryo development have been demonstrated by abundant studies, for instance, ET_B_ gene null mutation could result in embryonic death in GK rats [13]. In the vasculature, ETRs are closely linked with many disorders, particularly in the VSMC. Under pathophysiological conditions, the enhanced ET_A_ and ET_B2_ expressions in VSMC lead to dysregulated vascular tone and biological characteristics that are important for various cardiovascular disorders. The altered expressions of ETRs have been well-documented in patients and animal models of cardiocerebrovascular diseases, such as subarachnoid hemorrhage [14, 15], ischemic stroke [16, 17], and hypertension [18, 19]. Functional assessment revealed enhanced vasoconstriction mediated by ETR, inducing abnormalities of vascular tone [20]. ET_A_ and ET_B_ polymorphisms have been demonstrated to be associated with intracerebral hemorrhage [21].

To understand the association between ETR alteration and cardiovascular risk factors, for instance, cigarette smoking and dyslipidemia, we established an in vitro organ culture model and studied the effects of smoking particle extracts and LDL. It is intriguing to discover that ET_A_ and ET_B2_ expressions were upregulated by exposure to dimethyl sulfate-soluble smoking particles [6, 7], or to native LDL (through lipid peroxidation) [11]. These findings were supported by the results published by several groups [22, 23]. Various intracellular signals are responsible for the alteration of ETR, among which, the NF-*κ*B pathway plays a critical role. For example, the proinflammatory cytokine TNF-*α* upregulated ET_B2_ through activation of NF-*κ*B during organ culture of rat mesenteric arteries [5]. Whereas inhibition of NF-*κ*B significantly attenuated the upregulated ET_B2_ expression induced by cyclosporin A [24]. The above-mentioned studies established a fundamental link between risk factors, inflammation, and upregulation of ETRs in the vasculature.

To further elucidate the regulatory effects of inflammation on ETR alteration in VSMC in vivo, we intended to employ an animal model with simple immediate systemic inflammation. The LPS challenge model was chosen for the present study. Enhanced ET_A_ levels in renal arteries in LPS-treated portal hypertensive rats [25] and in cirrhotic rats [26] were reported. LPS-induced angiogenesis during chicken embryogenesis could be abolished by combined ET_A_ and ET_B_ receptor blockade, accompanied by a decrease in ET_A_ and ET_B_ expressions [27]. Additionally, ET_A_ and ET_B_ antagonists blocked oroxylin-A-induced vasoconstriction in endotoxemic mesenteric arteries (through intravenous injection of LPS), suggesting alteration of ETR [28]. In the present study, by intraperitoneal injection of LPS at a low dose (5 mg/kg body weight), we demonstrated not only the augmented mRNA/protein expressions of ET_A_ and ET_B_ but also the enhanced ETR-mediated vasoconstriction in VSMC in rat mesenteric arteries. Despite of the calculated pEC_50_ values that might not be reliable owing to lack of response to S6c in the NS group, the markedly increased *E*_Max_ values indicated the enhanced vasoreactivity to ETR agonists. This is in concern with a previous finding that intraperitoneal injection of a very high dose of LPS (20 mg/kg body weight) for 6 hrs induced pulmonary hypertension in rats [29]. Intraperitoneal administration of LPS could increase the TNF-*α* production by peritoneal macrophages and hence mediate severe liver damage in pancreatitis rats, leading to development of multiple organ failure [9, 10]. As a typical proinflammatory cytokine, TNF-*α* is known to be one of the most potent physiological inducers of the intracellular NF-*κ*B pathway [30]. Principally, the binding of a ligand to a cell surface receptor (like TNF-receptor) results in phosphorylation of IKK complex, which phosphorylates I*κ*B and subsequently leads to its ubiquitination and degradation by the proteasome, releasing NF-*κ*B to the nucleus to turn on target genes [31]. NF-*κ*B has been shown to be activated in all cells, where it regulates expression of diverse target genes and contributes to the pathogenesis of various diseases [32]. By our data, LPS challenge not only elevated the serum TNF-*α* level but also potently augmented ETR-mediated vasoconstriction and increased ETR mRNA/protein expressions, paralleled with activation of the intracellular NF-*κ*B pathway in VSMC in the mesenteric arteries. We speculate that the increased circulating TNF-*α* could result in activation of the NF-*κ*B pathway, and this might be at least partly responsible for the upregulation of ETRs in our model. The present data provided new insights into the effects of inflammation on the pathophysiological upregulation of ETRs in VSMC in vivo.

Clinical evaluations on ETR antagonists, including ambrisentan [33], macitentan [34, 35], and clazosentan [36, 37], confirmed the beneficial effects of ETR antagonism and further designated the combination of small-molecule epigenetic modulators and biologics like monoclonal ETR antibodies as novel pharmacological therapeutic strategies [38, 39]. Combining the evidence and clues that the pathophysiological upregulation of ETRs induced by inflammation has been observed under both in vitro and in vivo conditions, while the clinical application of ETR antagonists exert ameliorating effects, the alteration of ETRs could be a promising target in the treatment of cardiovascular disorders in clinic. Further studies are required to explore the in-depth mechanisms underlying the abnormalities in ETR expression, and clinical evaluations with emphasis on the indications of ETR antagonism in cardiovascular diseases could be taken into consideration.

## 5. Conclusion

By using a rat model of LPS-induced immediate systemic inflammation, we demonstrated the induction of ET_A_ and ET_B_ expressions at functional, mRNA, and protein levels in the VSMC in mesenteric arteries. The results provided new in vivo evidence showing inflammation-induced pathophysiological upregulation of ETRs and could be beneficial for understanding a possible mechanism of inflammation on exacerbating a vascular tone in the progression of vascular diseases.

## Data Availability

The data used to support the findings of this study are available from the corresponding author upon request.
